# Alternative AKT2 splicing produces protein lacking the hydrophobic motif regulatory region

**DOI:** 10.1371/journal.pone.0242819

**Published:** 2020-11-30

**Authors:** Guido Plotz, Laura A. Lopez-Garcia, Angela Brieger, Stefan Zeuzem, Ricardo M. Biondi

**Affiliations:** 1 Biomedizinisches Forschungslabor, Medizinische Klinik 1, Universitätsklinik Frankfurt, Frankfurt, Germany; 2 German Cancer Consortium (DKTK), German Cancer Research Center (DKFZ), Heidelberg, Germany; 3 Instituto de Investigación en Biomedicina de Buenos Aires (IBioBA)—CONICET—Partner Institute of the Max Planck Society, Buenos Aires, Argentina; International Centre for Genetic Engineering and Biotechnology, ITALY

## Abstract

Three AKT serine/threonine kinase isoforms (AKT1/AKT2/AKT3) mediate proliferation, metabolism, differentiation and anti-apoptotic signals. AKT isoforms are activated downstream of PI3-kinase and also by PI3-kinase independent mechanisms. Mutations in the lipid phosphatase PTEN and PI3-kinase that increase PIP3 levels increase AKT signaling in a large proportion of human cancers. AKT and other AGC kinases possess a regulatory mechanism that relies on a conserved hydrophobic motif (HM) C-terminal to the catalytic core. In AKT, the HM is contiguous to the serine 473 and two other newly discovered (serine 477 and tyrosine 479) regulatory phosphorylation sites. In AKT genes, this regulatory HM region is encoded in the final exon. We identified a splice variant of AKT2 (AKT2-13a), which contains an alternative final exon and lacks the HM regulatory site. We validated the presence of mRNA for this AKT2-13a splice variant in different tissues, and the presence of AKT2-13a protein in extracts from HEK293 cells. When overexpressed in HEK293 cells, AKT2-13a is phosphorylated at the activation loop and at the zipper/turn motif phosphorylation sites but has reduced specific activity. Analysis of the human transcriptome corresponding to other AGC kinases revealed that all three AKT isoforms express alternative transcripts lacking the HM regulatory motif, which was not the case for SGK1-3, S6K1-2, and classical, novel and atypical PKC isoforms. The transcripts of splice variants of Akt1-3 excluding the HM regulatory region could lead to expression of deregulated forms of AKT.

## Introduction

**Fig 1:**
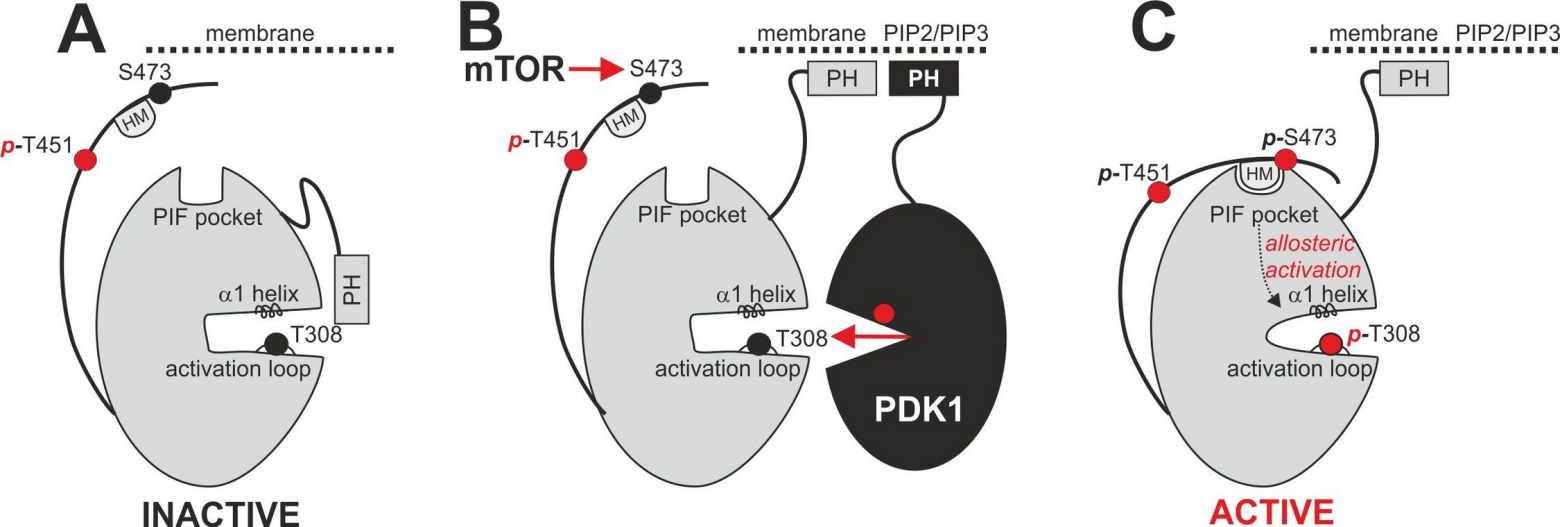
Activation of AGC kinases. A. The active site of inactive AGC kinases is inaccessible due to intramolecular shielding through the bound PH domain. B. Translocation to the membrane and binding of PIP2/PIP3 by the PH domain makes the kinase active site accessible for activation by PDK1 (which phosphorylates T308) and mTOR (which phosphorylates S473 at the hydrophobic motif HM). C. S473 phosphorylation triggers intramolecular binding of the hydrophobic motif (HM) regulatory site to the PIF pocket and further activation of the kinase by allosteric mechanisms.

AKT isoforms are activated downstream of PI3-kinase signaling and play both redundant as well as specific roles in signaling [1]. The phosphatidylinositol-3,4,5-triphosphate (PIP3) second messenger at the cell membrane triggers the recruitment of AKT to the membrane through its N-terminal PH-domain and enables its subsequent activation by its upstream kinases, PDK1 (*P*hosphoinositide-*d*ependent *k*inase-*1*, which phosphorylates Thr308/T309 in the activation loop–numbering residues in AKT1/AKT2, respectively) and mTOR (*m*echanistic *T*arget *o*f *R*apamycin, which phosphorylates Ser473/474 in the hydrophobic motif (HM)) (**Fig 1A and 1B**) [2]. The C-terminal extension also comprises the T451/T452 turn motif (also termed “zipper”) which is constitutively phosphorylated in AKTs [3]. Pathological elevation of PIP3 levels can either be conferred by mutations of PI3-kinase that enhance activity or by mutations in PTEN, the enzyme that dephosphorylates PIP3 and thus terminates the signal. Both types of mutations are frequently found in human cancers and enhance AKT phosphorylation and downstream signaling. More recently, it was found that additional phosphorylation sites modify AKT activity (serine 477 and also tyrosine 479 in AKT1) [4]. It has been widely considered that the activation loop phosphorylation activates AKT many fold while the HM phosphorylation further enhances the specific activity of AKT, although this is presently contested [4]. Once phosphorylated at the HM and at the activation loop, AGC kinases and in particular AKTs are activated by the intramolecular interaction of the HM to the PIF-pocket regulatory site and the concerted action of the activation loop and HM phosphorylation sites stabilizing the helix α1 and the active site in the proper active conformation (**Fig 1C**).

AKT activation in response to elevation of PIP3 levels involves the co-localization of AKT with its upstream kinase PDK1 (**Fig 1B**). This co-localization occurs via membrane binding and does not require a docking interaction between the HM and the PIF-pocket of PDK1, an interaction that is required for the phosphorylation of other substrates of PDK1, i.e. SGK, S6K, RSK, PKCs, etc. Although not required for activation of AKT by PDK1, in the absence of PIP3, the HM of AKT can indeed dock to the PIF-pocket of PDK1 [5]; this interaction has also been confirmed by the crystal structure of the complex between PDK1 and a polypeptide comprising the phosphorylated HM of AKT1 [6]. More recently, additional modes of AKT phosphorylation and activation, independently of PI3-kinase activation, have been described [4, 7, 8]. It is not known if the PI3-kinase independent activation of AKT requires the docking interaction of the HM to the PIF-pocket of PDK1.

The three AKT isoforms are encoded by three gene loci (AKT1, AKT2 and AKT3). AKT1 *null* mice show growth problems and perinatal lethality [9], while AKT2-knock-out animals develop insulin resistance and type 2 diabetes [10, 11]. AKT3 is predominantly expressed in brain and female tissues.

Alternative splicing is a powerful tool to generate different transcripts and hence distinct proteins from a single gene [12, 13]. Alternative splicing is abundant in humans and observed in most genes. Some genes have been shown to produce more “alternatively” spliced transcripts than the transcript(s) considered “regular” [14]. Changes in alternative splicing have been found to underlie human diseases [15].

Here we identified an alternatively spliced AKT2 transcript produced by activation of a cryptic exon located in the genes’ final intron. This generates a transcript that encodes an AKT2 isoform with an alternative C-terminus, which does not possess the HM (Ser474) and its contiguous Ser478 regulatory phosphorylation sites.

## Materials and methods

### Reagents, antibodies and cell lines

A 30-residue peptide representing the sequence of the cryptic exon of AKT2 (FREGFLEEEANVSAGRRNDVWDASNGRSMA) was generated by FPT Peptide Technologies, Berlin, Germany. The peptide was coupled to LPH (*Limulus polyphemus* Hemocyanine) and then used to produce polyclonal rabbit antiserum by BioGenes, Berlin, Germany. Rabbit polyclonal AKT2 antibody (#9272), anti-phospho Thr308 (#9275), anti-phospho Ser473 (#9271) and anti-phospho Thr451 (#9267) AKT antibodies were from Cell Signaling (MA, U.S.A.). The human embryonic kidney cell line HEK293 was maintained in DMEM supplemented with standard antibiotic and antimycotic solution and 10% FCS. Its identity was verified by genotyping by the DSMZ (Braunschweig, Germany). Transient transfections were performed with 5 μg of vector DNA and 20 μl of polyethyleneimine (1 mg/ml, ‘Max’ linear, 40 kDa; Polysciences, Warrington, PA) per 10 cm^2^ dish.

### Generation of vectors for expression and quantification

The cDNAs of AKT2 and its isoform were cloned into the eukaryotic expression vectors pcDNA3 and pEBG-2T (which contains a GST fusion tag at the N-terminus of the multiple cloning site). Suitable restriction sites (BamHI and NotI) were introduced by polymerase chain reaction, and the cDNAs were introduced in a two-step procedure, first by transferring the major fragment (BamHI and NotI) and subsequently a minor fragment (NotI) which is generated by an internal NotI restriction site within the AKT2 cDNA. The vectors were sequenced to confirm correct placement of the reading frames.

### Transcript level quantifications

For assessment of the transcript levels of AKT2 and its isoform in different tissues, we used the multiple tissue cDNA panels MTC I and MTC II from Clontech (#636742 LOT 5090205 and #636743 LOT6040176). These panels contain cDNA samples from organ tissues pooled from several individuals and normalized to at least four housekeeping genes.

TaqMan detection reagents (real-time quantitative PCR (qPCR) primers and probes) were generated via the FileBuilder software and synthesized by Applied Biosystems. For detection of the standard AKT2 transcript, a PCR product spanning exons 13–14 was used, while the PCR product for the AKT2-13a transcript covered exons 13 and the alternative exon 13a. The probes were spanning the exon boundaries to achieve specificity for cDNA. Control qPCR reactions with RNA samples which had not been reverse transcribed were performed to validate that no amplification occurs. qPCR reactions contained 15 μl total volume with TaqMan universal PCR master mix, the assay mixture containing primers and hydrolysis probe, and 1.5 μl sample. Cycling conditions were: 2 min 50°C, 10 min 95°C, 60 cycles with 15 s 95°C and 60 s 60°C. The StepOne 2.0 software was used to measure qPCR curves. Calibrations were performed for both transcripts using plasmid dilutions and confirmed comparable amplification efficiency in both detection reagents.

### Protein expression, immunoprecipitation and protein detection

AKT protein was produced by transient transfection of HEK293T cells. For this purpose, cells were plated into 10 cm dishes at 60% confluency. After three hours, AKT2 expression plasmids (or a GFP plasmid as transfection control) were transfected into HEK293T cells using 20μl of 1mg/ml PEI (linear, 25 kDa, #23966 Polysciences, USA) with 5 μg plasmid DNA in 1 ml DMEM. This transfection mixture was added to the cells, and whole cell extracts were prepared after 24 h and analyzed by western blotting. Visualization and quantification were performed using a LAS-4000 (Fuji).

For each immunoprecipitation, 22 μl AKT2-13a antibody solution were incubated with 4 μl Protein G Dynabead suspension (ThermoFischer Scientific, Waltham, MA) and HEK293 extracts either transfected with AKT2-13a (positive control, 30 μl) or with extract of untreated cells (1 ml) in a total volume of 1,5 ml in buffer A (50 mM Tris-HCl, pH 7.5, 0,1% β-Mercaptoethanol, 0,1 mM EGTA) at 4°C for 1 hour. Washing was performed with buffer A supplemented with 500 mM NaCl for three times.

### Bioinformatics

MaxEnt scores of AKT2 splicing sites were assessed in January 2019 using the appropriate web interfaces (http://genes.mit.edu/burgelab/maxent/Xmaxentscan_scoreseq.html) [16]. Data on splicing and exon usage of the AKT genes was retrieved from the genotype tissue expression project (GTEx) site [17].

### Protein purification and AKT2 kinase activity assay

GST-AKT2 wt and GST-AKT2-13a were expressed in 10 dishes (145 mm) of HEK293T cells as detailed above. Cells were harvested after 48 h in lysis buffer (50 mM Trizma pH 7.4, 0.27 M Sucrose, 1 mM Na-Ortho-Vanadate, 1 mM EDTA, 1 mM EGTA, 10 mM Na-ß-glycerolphosphate, 50 mM NaF, 5 mM Na-pyrophosphate, 1% Triton-x-100) and centrifuged (5.000 g, 15 min). The supernatant was incubated with 0.5 ml Glutathion-Sepharose 4B (GE Healthcare, Chicago, U.S.A), washed 4 times with 10 ml lysis buffer with 500 mM NaCl, 8 times with 10 ml buffer A (50 mM Trizma pH 7.4, 0.1 mM EGTA, 0,1% β-Mercaptoethanol), and once with 10 ml buffer A plus 0,26 M Sucrose. Elution was performed with buffer A supplemented with sucrose and 40 mM glutathione.

Kinase activity was assessed by incubating different amounts of purified AKT2 protein (100–1000 ng) in 20 μl reactions containing 0.1 mM KK-Crosstide (KKGRPRTSSFAEG; GenScript, Leiden, Netherlands), 50 mM Tris pH 7.4, 10 mM MgCl_2_, 0.1 mM ATP, 0.05 mg/mL BSA, 0.1% β-Mercaptoethanol in the presence of 60 nCi [^32^P]γATP (Perkin Elmer, Waltham, U.S.A.) for 30 minutes at room temperature. Reactions were terminated with 5 μl of a 1:25 dilution of phosphoric acid 88%. 4 μl of each reaction were applied to p81 paper (Whatman, Maidstone, U.K.). The paper was washed 4 times for 15 minutes with 50 ml of phosphoric acid (1:200 of 88%), dried and exposed to a phosphoimager IP screen overnight. Detection was performed with an LAS 9000 imager (Fuji, Japan). Quantifications were done using MultiGauge. The specific activity of GST-AKT2 was 3.5 U/mg, while the one of GST-AKT2 13a was 0.57 U/mg (one unit defined as the amount of activity that produces one nmol of product per minute).

## Results

### Alternative splicing in AKT transcripts

While attempting to clone AKT2 by PCR from a cDNA library from cancer-tissue, we unexpectedly identified a splice variant of AKT2 that lacked the HM regulatory site. Inspection of the gene indicated that this alternative mRNA resulted from activation of an alternative exon (exon 13a) located in intron 13 (IVS13+437; NG_012038.2:55788..55910) (**S1 Fig**). With a length of 123 bp, exon 13a has a typical exon size [18] and is framed by canonical splice sites. The quality of the splice sites, as determined by similarity to consensus splice sites, is below the average of the other AKT2 exons (**S2 Fig**). However, they support inclusion in the mRNA, similarly as has been found for another alternatively spliced gene before [19].

**Fig 2:**
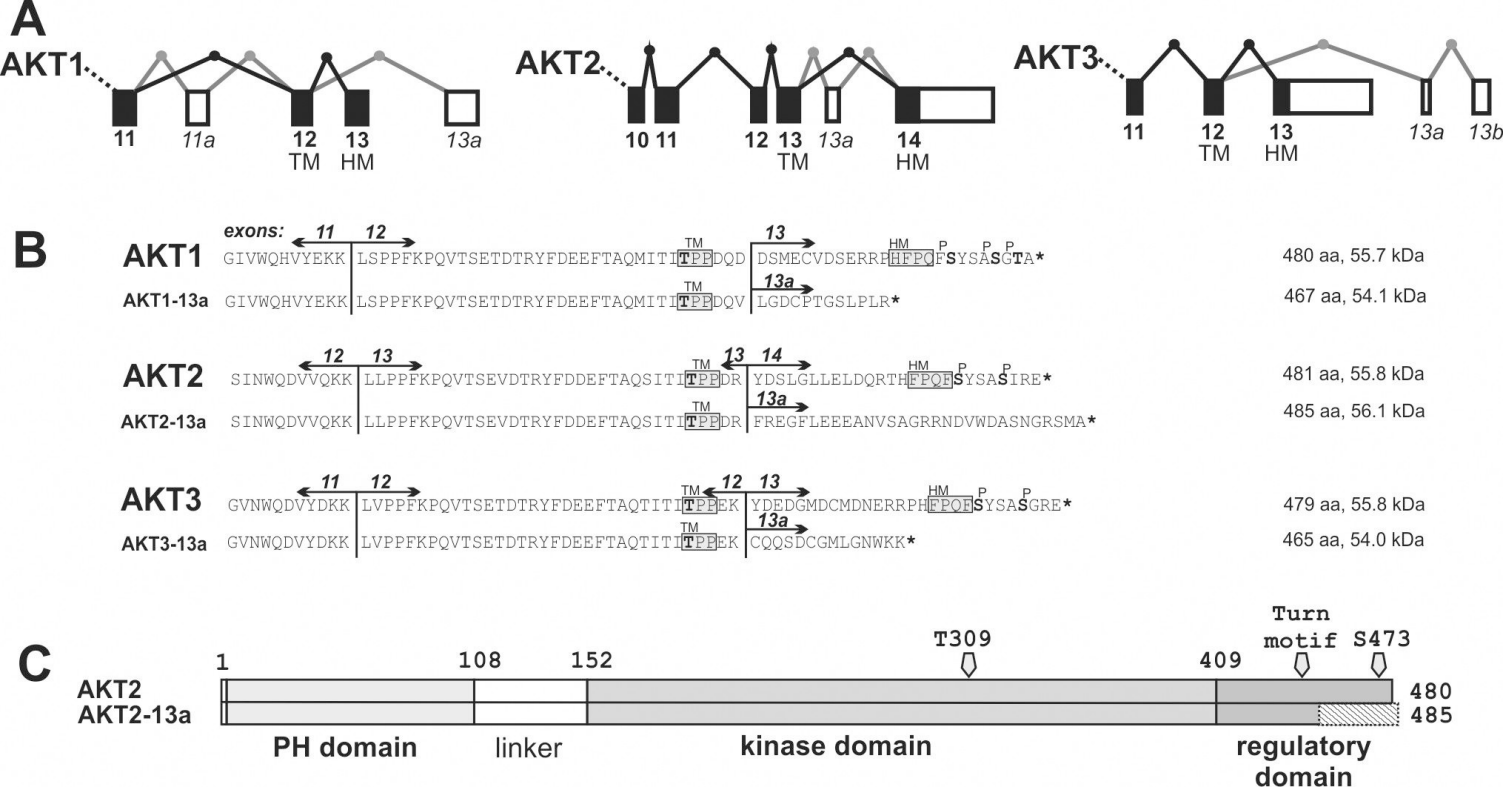
Splicing of AKT1-3 genes and predicted proteins of C-terminal splicing events. A. Exons included in the 3’-terminal reference transcripts of AKT1-3 are shown as black boxes connected by black lines, alternatively spliced exons are shown as white boxes with gray lines. Direction of transcription is from left to right. The two final exons contain the coding sequence of the turn motif (TM) and the hydrophobic motif (HM) phosphorylation sites. B. The C-terminal protein sequences of wildtype AKT1/AKT2/AKT3 as well as the predicted protein sequences of alternatively spliced transcripts are shown. Hydrophobic motif (HM), the turn motif (TM) and the phosphorylation sites are indicated. C. Representation of AKT2 protein (AKT2) and the AKT2-13a splice variant. The C-terminal fragment containing the HM is lost and replaced by an alternative C-terminus in AKT2-13a (hatched).

This finding prompted us to analyze the C-terminal splicing of AKT and other kinases containing HM regulatory sites in databases that assemble experimental data for splice variants in different tissues (**Fig 2A** and **S3 Fig**).

Alternative splicing affecting the C-terminal HM regulatory site has previously been observed in PKCβ, where two different protein isoforms (PKCβI and PKCβII) are generated by inclusion of two alternative final exons. In the case of PKCβ, the splice variants I and II both express PKCβ variants that have the TM and HM regulatory sites. Both PKCβ isoforms differ in their C-terminal tails: isoform I contains 50 residues and isoform II 52 residues, which both contain an HM motif and a sequence homology of 45% [20]. PKCβI and PKCβII exhibit different affinities for Ca^2+^, supporting the idea that the C-terminus of PKCβI and II also plays a role in the regulation of the C2 domain. However, splice-variant specific biological roles have not yet been revealed [21]. In contrast, other related AGC kinases such as PKCα, and all SGK, S6K and RSK isoforms did not show splicing of this regulatory domain in the databases of experimental splice variants. (**S3 Fig**).

Interestingly, all three AKT genes show alternative splicing that alters the 3’ end of the transcript structure. In all three AKT isoforms, the final exons of the genes include a part of the regulatory domain replacing the region of the hydrophobic motif (HM) phosphorylation sites (**Fig 2A**). Indeed, the AKT2 splice variant we found by cloning from a cDNA library had been experimentally observed and is present in the EST database.

AKT transcripts with alternative splicing in the 3’-end arise either by inclusion of an additional exon from the final intron (AKT2) or by usage of an alternative final exon located in the 3’ end of the gene (AKT1 and AKT3) (**Fig 2B**). It is noteworthy that the systematically identified splicings are of different types in all three AKT genes, suggesting that the spliced variants emerged independently after the duplication of the AKT gene. Experimental evidence therefore shows the existence of alternative transcripts in all three AKT kinases, which would result in proteins with altered C-termini (**Fig 2B**).

We selected the transcript AKT2-13a for further characterization. In AKT2-13a, the final 26 residues are substituted by 30 residues (**Fig 2C**). In contrast to the two alternative C-terminal PKC β isoforms, AKT2-13a does not encode a hydrophobic motif regulatory site in the C-terminus (**Fig 2B**).

### Detection and quantitation of the novel AKT2-13a transcript

**Fig 3:**
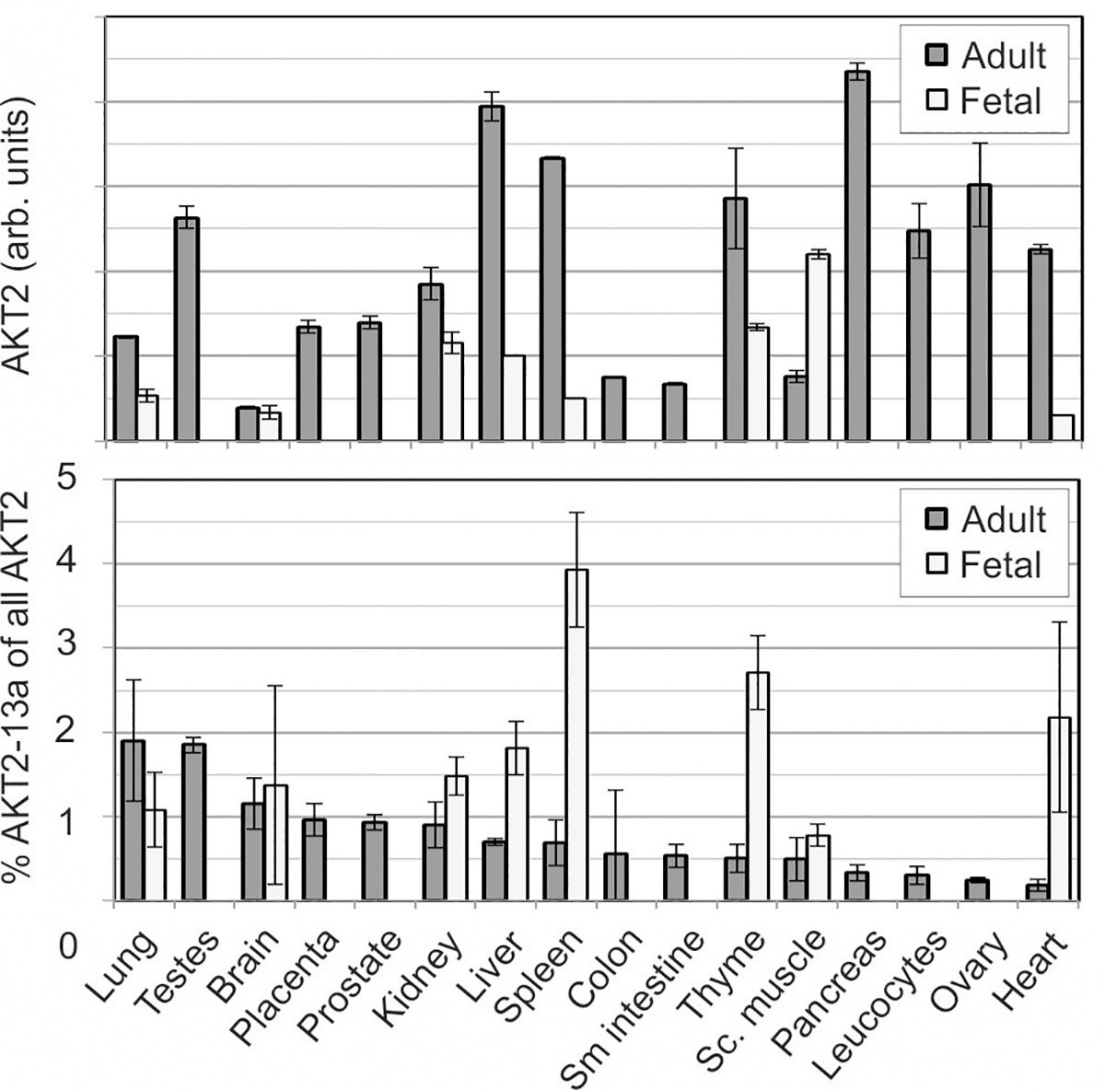
Quantitation of AKT2 and AKT2-13a in adult and fetal human tissues. Both AKT2 and AKT2-13a were detected by qPCR using specific primers and probes. Top diagram: Expression of AKT2 (standard transcript). Bottom diagram: Expression of the alternative transcript AKT2-13a in % of AKT2.

We generated a qPCR assay targeting either the standard transcript (comprising exons 13 and 14) or the alternative transcript (comprising exons 13 and 13a). Quantification in cell lines (Hela, HuH7, Hep3B, Hep4C, MCF7 and HEK293) demonstrated that the alternative transcript AKT2-13a represented between 0.8 and 3.1% of the regular AKT2 transcript. We furthermore investigated the relative quantity of mRNA in human healthy tissues of adult and fetal origins. The mRNA from regular AKT2 was most abundant in adult tissues of pancreas, liver, spleen and ovary, while it was much less expressed in brain, colon and small intestine, with a total difference of greater than tenfold (**Fig 3**). The quantitation of the alternative transcript AKT2-13a confirmed that it is low compared with the abundance of AKT2 in human tissues. However, there was a large difference between tissues: while AKT2-13a corresponded to 3.9% of mRNA of AKT2 in fetal spleen, it was not detectable in several other fetal tissues (**Fig 3**). On average, the AKT2-13a splice variant corresponded to 1.2% of the standard AKT2 gene, with the highest relative expression in fetal spleen (3.9%), thyme (2.6%) and heart (2.1%), followed by adult lung and testes.

We also tested the abundance of AKT2-13a in six cell lines (HEK293, Huh7, HepB3, HepC4, MCF). All cells expressed both wildtype AKT2 (**S4A Fig**) and the alternative variant AKT2-13a, which represented on average 2% of the wildtype AKT2 (0.8–3.1%), with highest relative expression in Hela cells (**S4B Fig**).

### The AKT2-13a transcript produces a protein that is phosphorylated at the activation loop and TM site but less active than AKT2

Since the alternative AKT2 transcript was readily detectable in cell lines and human tissues, we tested its ability to translate into a protein. The cDNA was cloned into two mammalian expression vectors, one of which generated a coding sequence for AKT2-13a as a fusion to GST.

**Fig 4:**
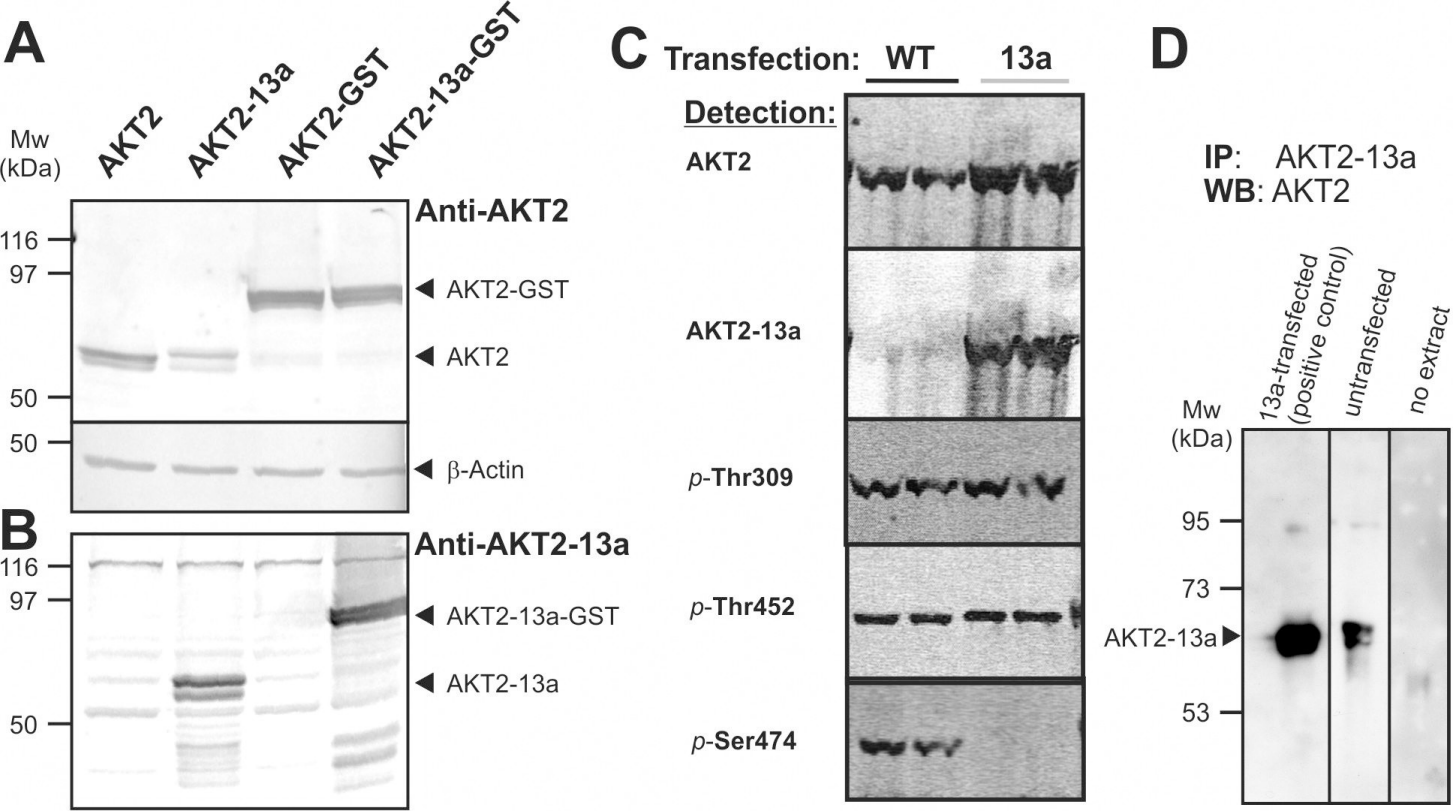
Protein detection of AKT2 and AKT2-13a. HEK293T cells were transfected with either pcDNA3 vectors containing cDNA coding for AKT2 and AKT2-13a or with pEBG-2T vectors which encode AKT2-GST fusion proteins. Cells were lysed after 24 hours and protein extracts were separated by SDS-PAGE followed by blotting and antibody detection with either a AKT2-specific antibody **(A)** or with an antibody generated to recognize the novel peptide sequence unique for AKT2-13a **(B)**. The AKT2-specific antibody produced double bands at the height of AKT2, the lower of which likely represent degradation products. **(C)**. AKT2 (“WT”) and AKT2-13a (“13a”) were transfected in HEK293 cells as detailed in Materials and Methods. Cells were harvested and protein extracts were prepared. Proteins were separated by SDS-PAGE. Immunoblotting was performed with detection of AKT2 or AKT2-13a and three antibodies specifically detecting the indicated phosphorylated residues (p-T309 is the activation loop; p-452 is in the TM; p-474 is in the HM). **(D)** HEK293T cells were either transfected with pcDNA-AKT2-13a (left, one dish) or left untransfected (right; thirty dishes). AKT2-13a was immunoprecipitated from protein extracts from these cells using the antibody directed against the unique C-terminal peptide of AKT2-13a. Washed precipitates were analyzed by SDS-PAGE and western blotting using AKT2 antibody detection.

For detection of the alternative protein isoform, a rabbit polyclonal antiserum was generated using a peptide with the novel C-terminal sequence (FREGFLEEEANVSAGRRNDVWDASNGRSMA). After transfection of AKT2 and the AKT2-13a isoform in HEK293 cells, both protein forms were detectable in a western blot of the whole cell extract by an antibody against the N-terminus of AKT2 (**Fig 4A**). As expected from the molecular weight calculation, the larger AKT2-13a ran higher on the gel than AKT2. The specific antibody for the isoform AKT2-13a detected major bands only in the lanes corresponding to cells transfected with plasmids coding for AKT2-13a (**Fig 4B**), further confirming the expression and identity of these signals. The same was true for the GST fusion proteins which were expressed in parallel. However, while the band strengths were identical in case of the GST fusion proteins, the expression of AKT2-13a was lower than that of standard AKT2 (**Fig 4A**). We then asked whether the splice variant AKT2-13a was phosphorylated in our preparations. Interestingly, we found that AKT2-13a was phosphorylated at Thr309 at the activation loop, and at Thr452, the TM phosphorylation site at similar levels as AKT2 (**Fig 4C**). As expected, no signal was present for phospho-Ser474 in AKT2-13a preparations since this residue is lacking in the alternatively spliced AKT2-13a isoform.

In order to assess endogenous AKT2-13a protein, we investigated its presence in extracts of untransfected cells, but the anti-serum developed to the AKT2-13a specific sequence did not detect endogenous protein in cell extract. However, we detected the endogenous expression of AKT2-13a with the new anti-serum by western-blot after immunoprecipitation of AKT2 using antibodies that recognize the PH domain (**Fig 4D**).

**Fig 5:**
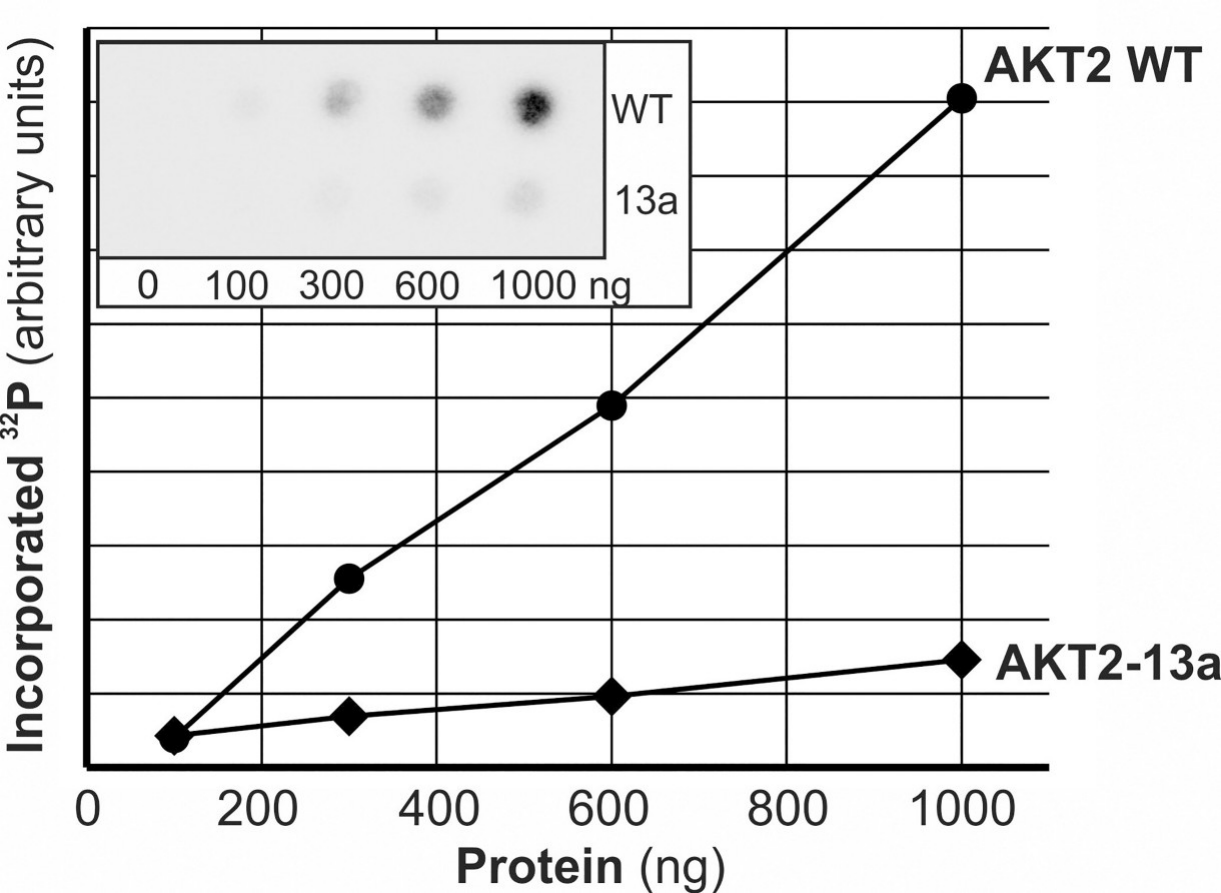
Kinase activity of GST-AKT2-13a in comparison to GST-AKT2 wt. Kinase activity of GST-AKT2-13a was assessed in comparison to wildtype GST-AKT2 after purification of both proteins (**S5 Fig**) by measuring the phosphorylation of Crosstide of different protein amounts (100, 300, 600 and 1000 ng) as detailed in Materials and Methods. Incorporation of ^32^P into Crosstide was scored by phosphoimager quantification of radioactive signal (inlay in graph).

In order to assess the kinase activity of the variant AKT2 isoform, we purified both normal GST-AKT2 and GST-AKT2-13a protein to >95% (**S5 Fig**). Kinase activity on the peptide substrate Crosstide was assessed at different protein concentrations and was much lower for AKT2-13a than for wildtype AKT2, with an average relative activity of 16% (**Fig 5**).

## Discussion

The general model to explain the mechanism of activation of AKT downstream of PI3-kinase, was depicted almost two decades ago. Interestingly, over the last few years new important aspects on the mechanism of activation of AKT were described, most notably, the PI3-kinase-*independent* activation. The C-terminal region of AKT, comprising the TM and HM sites play a key regulatory role in the models of AKT activation. The HM directly binds to the PIF-pocket regulatory site on the small lobe of the catalytic domain, while the phosphorylated TM site binds to a phosphate-binding site on top of the ATP-binding site, and supports this intramolecular binding of the HM to the PIF-pocket in the active conformation (**Fig 1**). While the role of the HM on the active structure of AKT is well established, the possible role of the HM in the docking interaction with the upstream kinase PDK1 in the PI3-kinase independent activation or in the role of the HM in the inactive structure of AKT is not known. In this context, we here highlight the existence of splicing variants that replace the C-terminal region of all three isoforms of AKT which may differ in their regulation. While a possible physiological role of the AKT2-13a splice variant is not known, the fact that it appeared phosphorylated at the activation loop (T309) and TM sites suggests that the AKT2-13a is recognized by upstream kinases. The specific activity of AKT2-13a was 6 times lower, indicating that the amino acid sequence replacing the hydrophobic motif induced AKT2-13a to behave as expected by AKT2 that is not phosphorylated at the hydrophobic motif. AKT2-13a is well folded and has activity; therefore its overexpression could deregulate downstream signaling pathways.

In AKT1, AKT2 and AKT3 genes, the resulting AKT splice variant protein isoforms would retain the major part of the protein: PH-domain and kinase domain remain identical (**Fig 2B**), while the C-terminal regulatory domain would be altered such that the coding sequence for the hydrophobic motif and its adjacent phosphorylation sites are deleted. While the alternative variants of AKT1 and AKT3 result in proteins with significantly altered molecular weight (**Fig 2B**), AKT2-13a is most likely to evade detection in standard analyses. However, it is frequent that researchers overlook additional lower intensity bands of protein kinases observed by western-blot of crude extracts. Minor bands could be due to post-translational modifications, but could also originate from the expression of splicing variants. We suggest that proteomic studies should include the C-terminal AKT splice variants in their analysis.

Besides lacking the HM and the 473 phosphorylation site, AKT2-13a additionally lacks the terminal serine 477 and tyrosine 479 phosphorylation sites, suggesting that it will also lack regulation mediated by these sites [4]. The HM of AKT is not bound to the PIF-pocket in the inactive conformation. On the other hand, the non-phosphorylated hydrophobic motif (Phe-xx-xx-Phe-Ser-Tyr) is unlikely to be free in solution and could have binding partners that are lost in all AKT splice variants. AKT isoforms have been described to bind to CTMP via its HM sequence, conferring a negative regulation [22]; such regulation is also expected to be missing in AKT2-13a.

Interestingly, while the regulation of kinases mediated by the HM is widely present in AGC kinases, alternative transcripts that lack the HM sequence cannot be observed in many related kinases (SGK1-3, S6K1/2, PKCα/⍳/ζ). In PKCβ, two alternative final exons exist, but both contain hydrophobic motifs and their phosphorylation sites. Both protein isoforms (PKCβ I and PKCβ II) differ in their C-terminal 50/52 amino acids, which share 45% homology, and have been found to be practically identical in physical and kinetic properties [23]. No differences in biological role(s) has as yet been attributed to these isoforms, which may be attributable to the partly redundant and overlapping functions of PKC isoforms.

A qPCR specifically distinguishing the alternative transcript AKT2-13a from the normal AKT2 demonstrated a significantly lower relative abundance of the alternative transcript AKT2-13a, which was on average 1–2% of standard AKT2. Highest levels were observed in fetal tissues (roughly 4% in fetal spleen). The level of relative expression of the AKT2-13a transcript is low in comparison to the standard transcript. It may be argued that the expression of the AKT2-13a transcript may be due to the error rate of the mRNA and splicing machinery that may be related to the overall expression of the AKT2 gene. However, it is noteworthy that there is up to 5-fold difference in the relative expression between different tissues, suggesting that there is a tissue-dependent differential splicing of AKT2-13a. Moreover, it is at least curious that all three AKT isoforms present alternative splicing forms lacking the HM while other AGC kinases do not show such “errors”.

The novel AKT2-13a form may have a biological function, possibly during embryogenesis, since its expression was higher in fetal tissues. Alternatively, it is possible that it is a biologically non-relevant side-product of the splicing. Such side-products arise in normal gene transcription, partly due to errors in splice site recognition [14]. In germline mutation detection methods based on cDNA sequencing, up to 10–25% abnormally spliced products routinely are considered normal “errors” [24]. The considered intrinsic error rate of the splicing machinery thus makes it difficult to distinguish potentially relevant transcripts from the noise of by-product “errors” [25].

When overexpressed, AKT2-13a accumulated less than the wildtype protein, suggesting a compromised protein stability. AKT proteins depend on conformational integrity which is in part conferred by phosphorylation of the TM phosphorylation site T452 [26]. While T452 was present and phosphorylated in AKT2-13a, its stabilizing effect probably was compromised by the alternative C-terminal sequence. Interestingly, the C-terminal GST fusion proteins displayed identical stabilities with wildtype AKT2 and AKT2-13a, suggesting that the C-terminal protein extension reverts the destabilizing effect of the non-regular sequence. GST-AKT2-13a displayed a strongly reduced kinase activity in comparison to GST-AKT2. AKTs have a basal activity when they are phosphorylated at the turn motif and activation loop sites and further reach maximal specific activities upon phosphorylation of the hydrophobic motif. The lower *in vitro* specific activity of GST-AKT2-13a indicates that the alternative C-terminal sequence does not bind to the PIF-pocket, or, alternatively, if it binds at the site, it does not promote the conformational changes that activate the kinase. Moreover, the decreased activity of this splice variant further confirms the biological relevance of the hydrophobic motif in the regulation of the specific activity of AKT2.

Taken together, we have investigated an alternative splice variant of human AKT2 that is predicted to encode a protein with a different C-terminal regulatory tail lacking the hydrophobic motif regulatory site. The alternative exon shows no evolutionary conservation. While the finding that all AKT isoforms form aberrant transcripts with a different C-terminal regulatory region suggest a potential biological role, our current observations do not unveil a physiologically relevant regulatory contribution. A physiological or pathophysiological relevance of the existence of AKT isoforms with a differential C-terminal regulatory region remains to be established.

## Supporting information

S1 Fig Location and sequence of the alternative exon 13a. The AKT2 gene is shown with all exons that are spliced into the regular reference sequence, with the exon numbers indicated above the exons. The alternative exon is located in the middle of intron 13 IVS13+437; NG_012038.2:55788..55910. The splicing sites (with the relevant bases) are indicated by boxed sequence, the invariable dinucleotides (AG..GT) are printed in fat and underlined. The exon sequence is printed in capitals, non-coding sequence in small lettering.
(DOCX)

S2 Fig MaxEnt scores for the AKT2 gene. MaxEnt scores are displayed for all 14 regular AKT2 exons (1–14) as well as for the cryptic exon 13a. 5’-splice sites (boxes) and 3’-splice sites (circles) of each exon have been connected with a line representing the exon sequence.
(DOCX)

S3 Fig Location of the *hydrophobic motif* and splicing in several kinases. Direction of transcription is from left to right. Boxes indicate (alternative) exons, (alternative) splicing events that have been observed in the genes are indicated by the lines with dots that show which exons have been observed to be spliced together. The arrow with HM indicates the locations of the hydrophobic motif. The figures have been adopted from the overview of alternative splicing in different tissues from the GTEx portal.
(DOCX)

S4 Fig Expression of AKT2 and AKT2-13a in cell lines. Both AKT2 and AKT2-13a were detected by qPCR using specific primers and probes. **A**: expression of AKT2 (standard transcript) relative to the housekeeping gene GAPDH. **B**: expression of the alternative transcript AKT2-13a in % of AKT2.
(DOCX)

S5 Fig GST-AKT2 and GST-AKT2-13a were expressed in HEK293T cells as detailed in materials and methods. **A**. Both proteins were visible in Coomassie staining of cell extract after SDS-PAGE. **B**. Western blot detection using anti-GST antibody of the cell extract. **C**. Coomassie staining of an SDS-PAGE of purified GST-AKT2 and GST-AKT2-13a (1 μg and 5 μg each, respectively).
(DOCX)

S1 File (DOCX)
