# Supplementary material for: Alternative AKT2 splicing produces protein lacking the hydrophobic motif regulatory region
Source: PLoS One. 2020 Nov 30;15(11):e0242819. doi: 10.1371/journal.pone.0242819 (PMC7703976; doi:10.1371/journal.pone.0242819)
Supplement: S1 Fig — The AKT2 gene is shown with all exons that are spliced into the regular reference sequence, with the exon numbers indicated above the exons. The alternative exon is located in the middle of intron 13 IVS13+437; NG_012038.2:55788..55910. The splicing sites (with the relevant bases) are indicated by boxed sequence, the invariable dinucleotides (AG..GT) are printed in fat and underlined. The exon sequence is printed in capitals, non-coding sequence in small lettering. (DOCX) [file pone.0242819.s001.docx]

**S1 Fig**. Location and sequence of the alternative exon 13a.


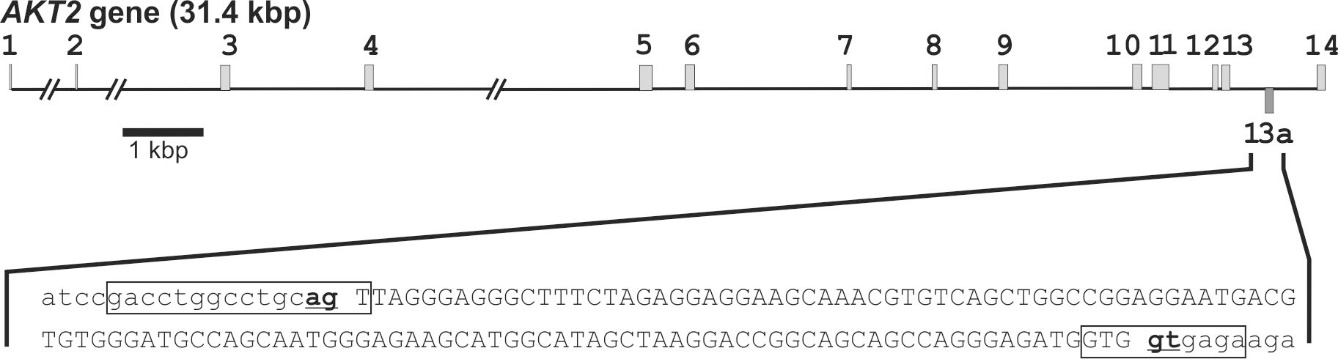


**S1 Fig**. The AKT2 gene is shown with all exons that are spliced into the regular reference sequence, with the exon numbers indicated above the exons. The alternative exon is located in the middle of intron 13 IVS13+437; NG_012038.2:55788..55910. The splicing sites (with the relevant bases) are indicated by boxed sequence, the invariable dinucleotides (AG..GT) are printed in fat and underlined. The exon sequence is printed in capitals, non-coding sequence in small lettering.
