# Supplementary material for: Alternative AKT2 splicing produces protein lacking the hydrophobic motif regulatory region
Source: PLoS One. 2020 Nov 30;15(11):e0242819. doi: 10.1371/journal.pone.0242819 (PMC7703976; doi:10.1371/journal.pone.0242819)
Supplement: S2 Fig — MaxEnt scores are displayed for all 14 regular AKT2 exons (1–14) as well as for the cryptic exon 13a. 5’-splice sites (boxes) and 3’-splice sites (circles) of each exon have been connected with a line representing the exon sequence. (DOCX) [file pone.0242819.s002.docx]

**S2 Fig. MaxEnt scores for the AKT2 gene.**


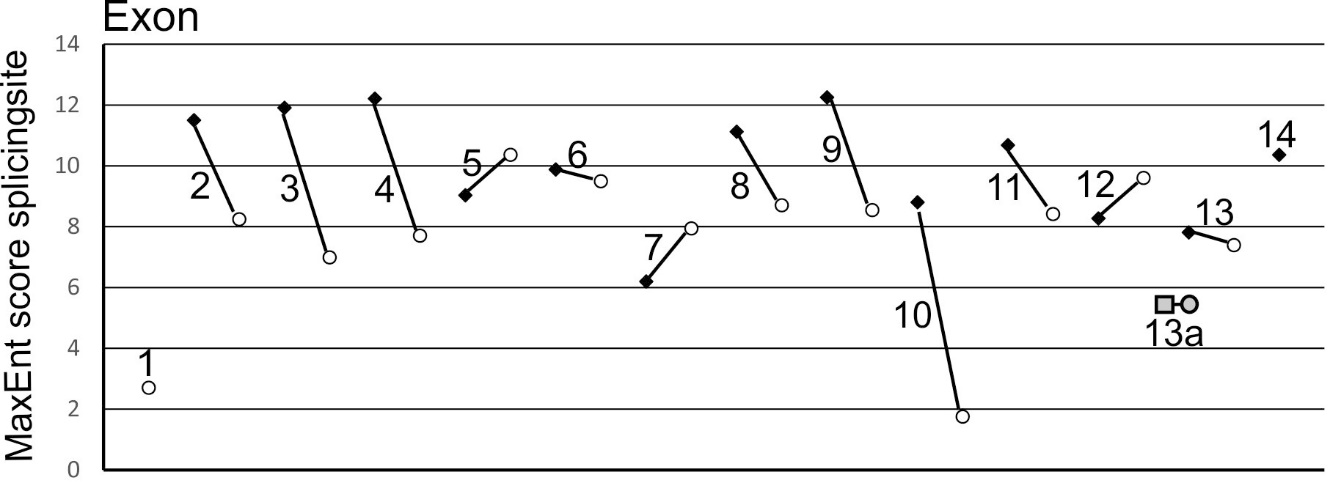


**S2 Fig**. MaxEnt scores are displayed for all 14 regular AKT2 exons (1-14) as well as for the cryptic exon 13a. 5’-splice sites (boxes) and 3’-splice sites (circles) of each exon have been connected with a line representing the exon sequence.
