# Supplementary material for: Alternative AKT2 splicing produces protein lacking the hydrophobic motif regulatory region
Source: PLoS One. 2020 Nov 30;15(11):e0242819. doi: 10.1371/journal.pone.0242819 (PMC7703976; doi:10.1371/journal.pone.0242819)
Supplement: S3 Fig — Direction of transcription is from left to right. Boxes indicate (alternative) exons, (alternative) splicing events that have been observed in the genes are indicated by the lines with dots that show which exons have been observed to be spliced together. The arrow with HM indicates the locations of the hydrophobic motif. The figures have been adopted from the overview of alternative splicing in different tissues from the GTEx portal. (DOCX) [file pone.0242819.s003.docx]

**S3 Fig. Location of the *hydrophobic motif* and splicing in several kinases**


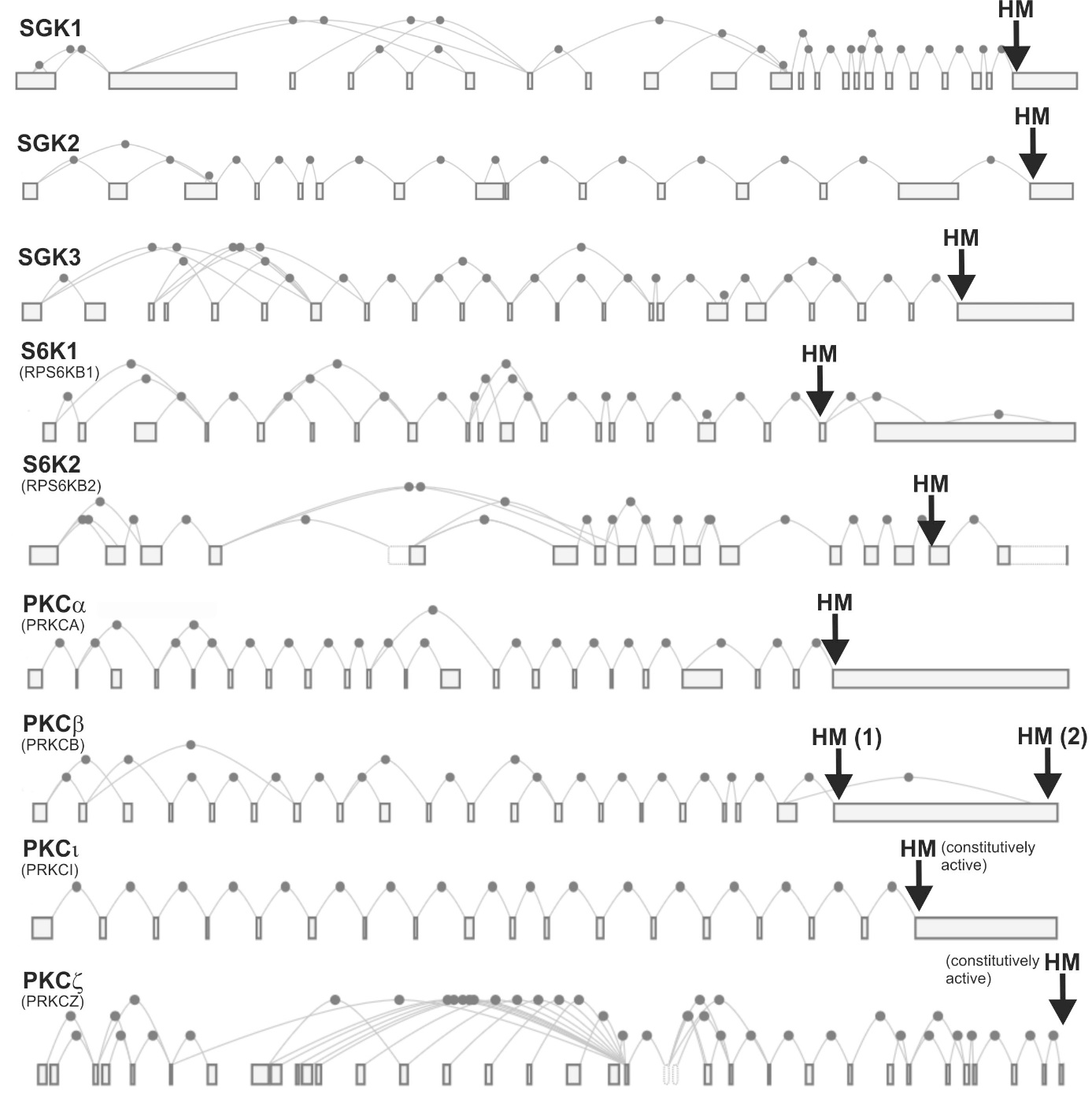


**S3 Fig**: Direction of transcription is from left to right. Boxes indicate (alternative) exons, (alternative) splicing events that have been observed in the genes are indicated by the lines with dots that show which exons have been observed to be spliced together. The arrow with HM indicates the locations of the hydrophobic motif. The figures have been adopted from the overview of alternative splicing in different tissues from the GTEx portal.
