# Supplementary material for: Alternative AKT2 splicing produces protein lacking the hydrophobic motif regulatory region
Source: PLoS One. 2020 Nov 30;15(11):e0242819. doi: 10.1371/journal.pone.0242819 (PMC7703976; doi:10.1371/journal.pone.0242819)
Supplement: S4 Fig — Both AKT2 and AKT2-13a were detected by qPCR using specific primers and probes. A: expression of AKT2 (standard transcript) relative to the housekeeping gene GAPDH. B: expression of the alternative transcript AKT2-13a in % of AKT2. (DOCX) [file pone.0242819.s004.docx]

**S4 Fig. Expression of AKT2 and AKT2-13a in cell lines.**

**
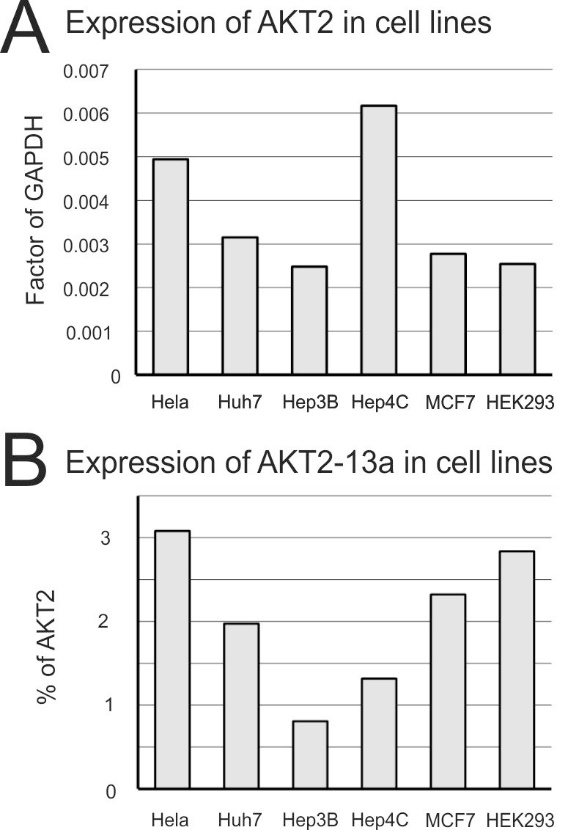
**

**S4 Fig:** Both AKT2 and AKT2-13a were detected by qPCR using specific primers and probes. **A**: expression of AKT2 (standard transcript) relative to the housekeeping gene GAPDH. **B**: expression of the alternative transcript AKT2-13a in % of AKT2.
