# Supplementary material for: Alternative AKT2 splicing produces protein lacking the hydrophobic motif regulatory region
Source: PLoS One. 2020 Nov 30;15(11):e0242819. doi: 10.1371/journal.pone.0242819 (PMC7703976; doi:10.1371/journal.pone.0242819)
Supplement: S5 Fig — A. Both proteins were visible in Coomassie staining of cell extract after SDS-PAGE. B. Western blot detection using anti-GST antibody of the cell extract. C. Coomassie staining of an SDS-PAGE of purified GST-AKT2 and GST-AKT2-13a (1 μg and 5 μg each, respectively). (DOCX) [file pone.0242819.s005.docx]

**S5 Fig. Expression and purification of GST-AKT2 and GST-AKT2-13a.**


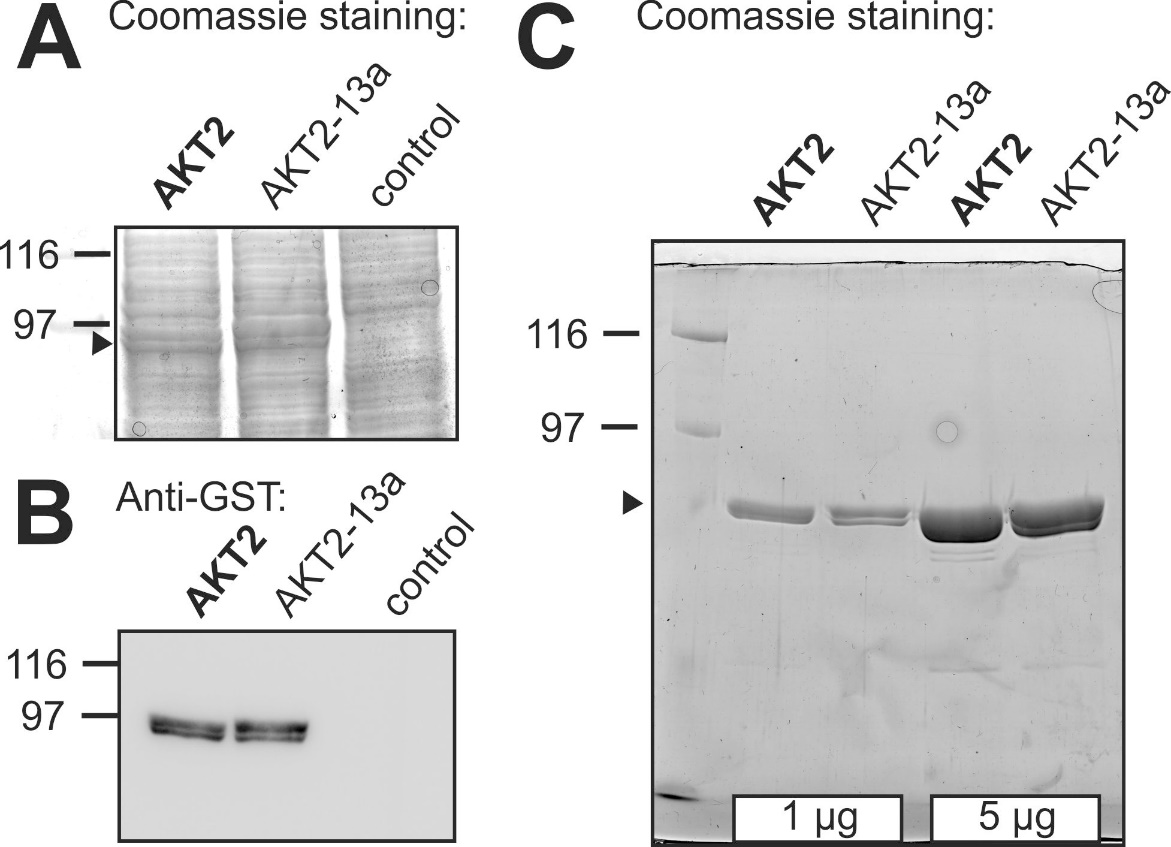


**S5 Fig:** GST-AKT2 and GST-AKT2-13a were expressed in HEK293T cells as detailed in Materials and Methods. **A**. Both proteins were visible in Coomassie staining of cell extract after SDS-PAGE. **B**. Western blot detection using anti-GST antibody of the cell extract. **C**. Coomassie staining of an SDS-PAGE of purified GST-AKT2 and GST-AKT2-13a (1 µg and 5 µg each, respectively).
