# Supplementary material for: Alternative AKT2 splicing produces protein lacking the hydrophobic motif regulatory region
Source: PLoS One. 2020 Nov 30;15(11):e0242819. doi: 10.1371/journal.pone.0242819 (PMC7703976; doi:10.1371/journal.pone.0242819)
Supplement: S1 File — (DOCX) [file pone.0242819.s006.docx]

ORIGINAL UNCROPPED AND UNADJUSTED IMAGES


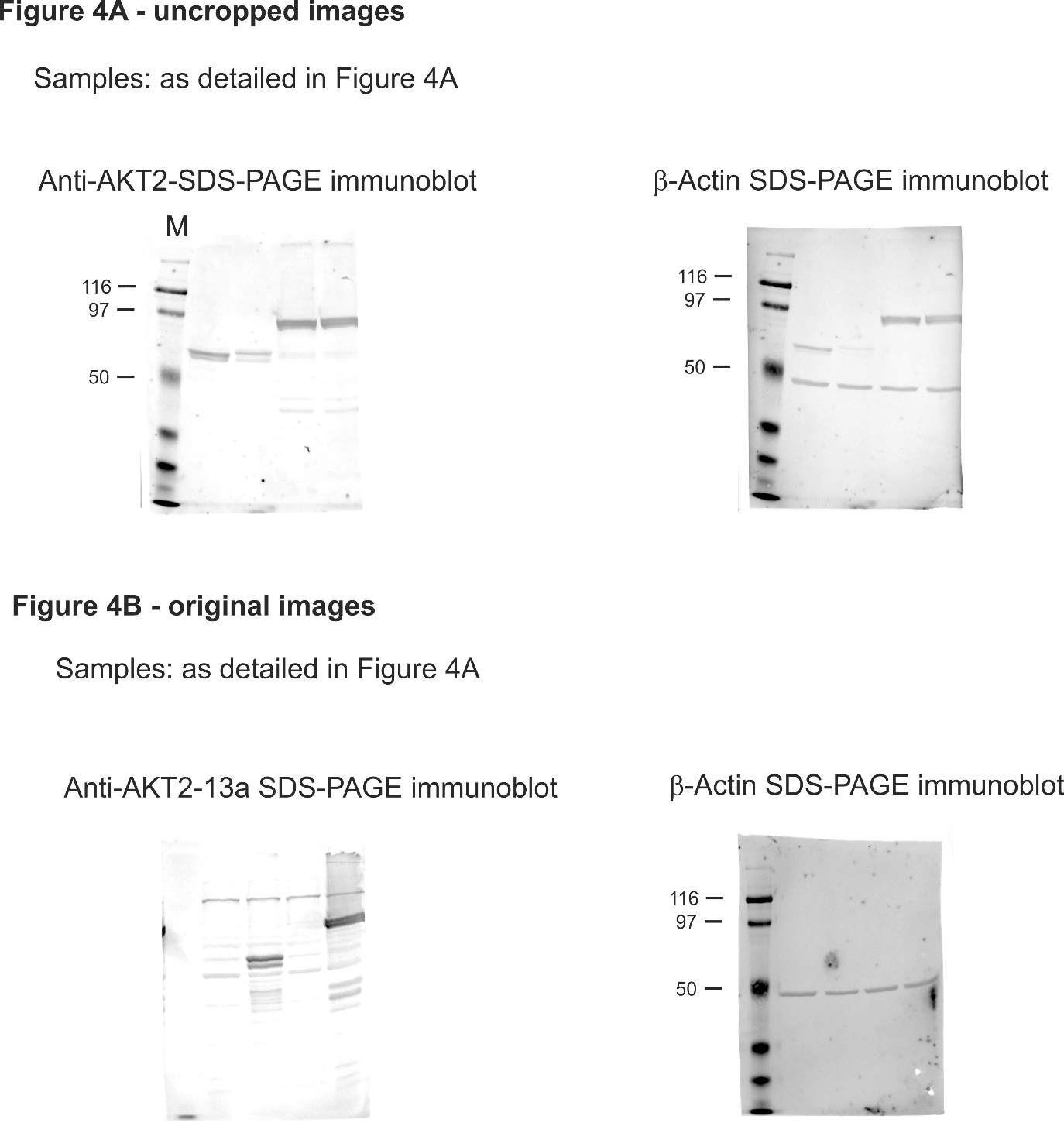


ORIGINAL UNCROPPED AND UNADJUSTED IMAGES


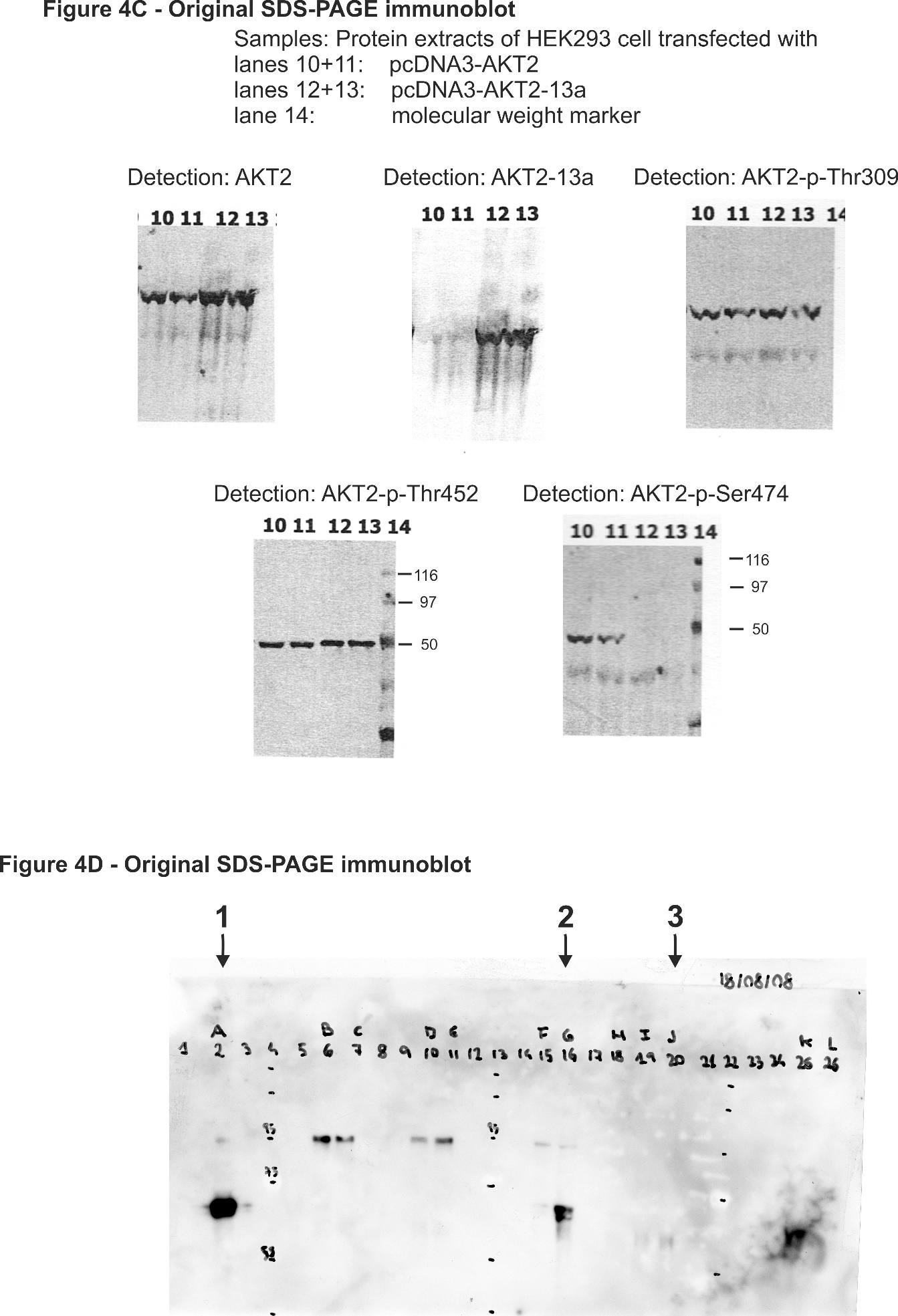


ORIGINAL UNCROPPED AND UNADJUSTED IMAGES

**
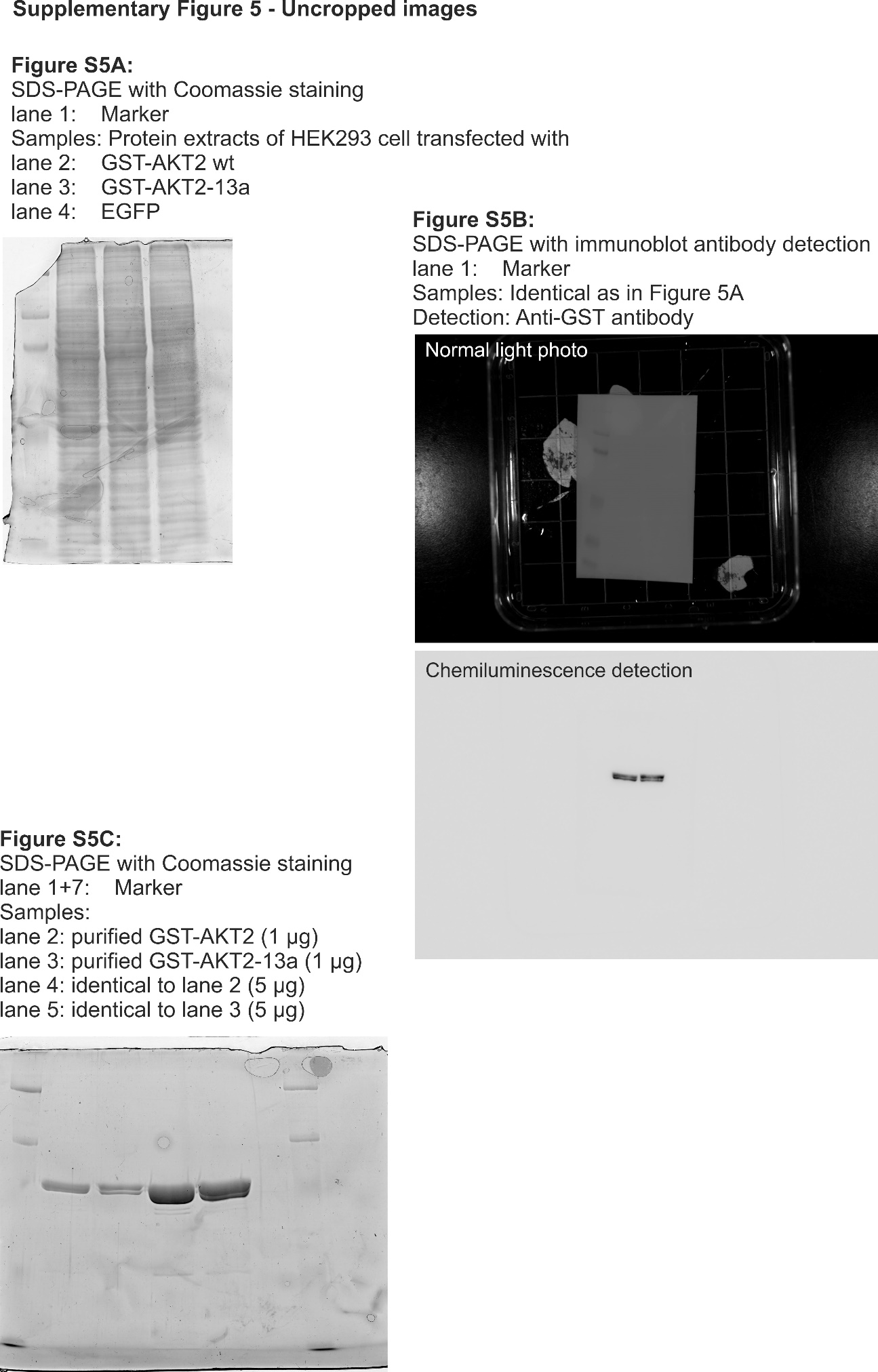
**
